# Depressive symptoms before and after Parkinson’s diagnosis—A longitudinal analysis

**DOI:** 10.1371/journal.pone.0272315

**Published:** 2022-07-29

**Authors:** Shengfang Song, Zhehui Luo, Chenxi Li, Xuemei Huang, Eric J. Shiroma, Eleanor M. Simonsick, Honglei Chen

**Affiliations:** 1 Department of Epidemiology and Biostatistics, College of Human Medicine, Michigan State University, East Lansing, MI, United States of America; 2 Department of Neurology, Hersey Medical Center, Pennsylvania State University, Hersey, PA, United States of America; 3 Intramural Research Program of the National Institute on Aging, Baltimore, MD, United States of America; North Karelia Central Hospital, FINLAND

## Abstract

**Background:**

Depression is common in Parkinson’s disease (PD). It is however unclear when and how depressive symptoms develop and progress in the course of PD development.

**Objective:**

To assess how depressive symptoms evolve in PD, using repeated measures.

**Methods:**

In 2994 older adults, ages 70–79 years, depressive symptoms were assessed 8 times over 11 years using the 10-item Center for Epidemiologic Studies Depression Scale (CESD-10). For each PD patient at each time point, we calculated the difference between CESD-10 score and its expected value estimated based on data from individuals without PD, and then realigned the time scale in reference to the year of PD diagnosis. We examined longitudinal changes in CESD-10 scores before and after PD diagnosis using a joint modeling approach to account for competing risks of non-participation and death.

**Results:**

A total of 79 PD patients were identified at enrollment or during the follow-up, with repeatedly assessed depressive symptom data up to 9 years before and after PD diagnosis. We found a monotonic trend of increasing CESD-10 score in PD patients throughout the observational period (p = 0.002). The observed scores became higher than expected approximately 7 years before PD diagnosis and significantly different 1 year before PD diagnosis.

**Conclusions:**

Increasing depressive symptomatology appears to precede PD diagnosis by a few years.

## Introduction

Depressive symptoms are among the most common nonmotor symptoms of Parkinson’s disease (PD) [1, 2], affecting about 35% of patients [3]. Depression jeopardizes daily functioning [4] and quality of life of PD patients [5, 6], and may lead to earlier initiation of dopaminergic therapy [4] and faster physical and cognitive deterioration [2, 7, 8]. Further, depression may develop in prodromal PD. A good understanding of the temporal relationship between depression and PD clinical onset may inform the disease’s natural history and early identification. However, this timeline remains obscure mainly due to the lack of longitudinal repeated measurements of depressive symptoms in the course of PD development. By capitalizing on annually or biennially assessed depressive symptoms in a community-based biracial cohort over 11 years, we examined how depressive symptoms develop and progress before and after PD diagnosis.

## Materials and methods

### Study design and population

The Health, Aging, and Body Composition (Health ABC) study was designed to study risk factors for functional declines in older adults, especially changes in body composition, behavioral and physiological conditions in the context of aging [9]. The study recruited 3075 well-functioning older adults (age 70–79, 51.4% women, 41.6% blacks) in 1997–1998 living in Pittsburgh, Pennsylvania, and Memphis, Tennessee. Inclusion criteria included 1) no difficulty in walking 1/4 mile or climbing up 10 steps; 2) no mobility-related difficulty in performing everyday tasks; 3) no intention to move out of the study area in the next three years. Exclusion criteria included 1) active cancer treatment in the past three years; 2) current participation in a lifestyle intervention trial. Participants enrolled in the study by completing the Year 1 baseline clinical visit from April 1997 to June 1998. Their health and survival were monitored for up to 17 years with annual or biennial clinical visits, semiannual/quarterly phone calls, and hospitalization and death surveillance. All participants provided written informed consent, and the study protocol was approved by the Institutional Review Boards at the University of Pittsburgh and the University of Tennessee–Memphis. This specific secondary data analysis was IRB exempted as non-human research by the Michigan State University.

### Assessment of depressive symptoms

Depressive symptoms were assessed eight times in clinical visits year 1, 3, 4, 5, 6, 8, 10, and 11 using the 10-item Center for Epidemiologic Studies Depression Scale (CESD-10) [10]. CESD-10 asks participants to report the frequency of 10 depressive symptoms that they experienced in the past week, ranging from “rarely” (0) to “most of the time” (4). The scoring scheme is reversed for symptoms of positive emotion. The total score adds to a maximal of 30, with a higher score for CESD-10 representing more severe depressive symptoms [11]. In the assessment, we coded “don’t know” or “refused” as missing. If two or more items were missing (11.6%), we coded the overall CESD-10 score as missing. If there is only one missing item (1.2%), we prorated the score according to completed items. As the CESD-10 weighs more on depressed mood and thinking but less on somatic symptoms [12], it is suitable for assessing depressive symptoms longitudinally across a range of PD disease severity [12]. We analyzed the CESD-10 score as a continuous variable because we aimed to assess the spectrum severity of depressive symptoms longitudinally as PD develops and progresses.

### PD ascertainment

In 2015, we conducted a retrospective PD case adjudication by comprehensively reviewing relevant medical data collected during the cohort’s health follow-up as detailed previously [13]. Briefly, we first identified a total of 156 potential PD cases if any of the following was true: 1) use of antiparkinsonian medications (carbidopa/levodopa, dopamine agonists, monoamine oxidase B inhibitors, amantadine, or anticholinergic drugs) at any available clinic visit; 2) self-reported PD diagnosis; 3) adjudication of PD as the cause of hospitalization; 4) PD as the adjudicated cause of death. For each potential case, two experienced movement disorder specialists independently reviewed relevant data over the entire follow-up, accounting for the number of independent sources that indicated a PD diagnosis, internal consistency within each source, and evidence against PD diagnosis. The final diagnostic adjudications were made by consensus. Of the 156 potential cases identified, 81 were confirmed as having PD. Of the rest, 58 had an uncertain determination and thus were excluded from the analysis; the other 17 were adjudicated as no PD cases and thus were retained in the study as controls. We further defined the year of diagnosis as either the first year that PD medication or diagnosis was reported or, for PD first identified by hospitalization or death, the midpoint between the first identification and the previous year of the medical survey without PD medication use. In the analysis, we used the year of PD diagnosis as the reference time point, similar to what we published previously [14, 15]. For each PD case at each clinical visit, we calculated the number of years in reference to the time of PD diagnosis by subtracting the calendar year of PD diagnosis from the year of a clinic visit. For example, if a case got a PD diagnosis in 2002 and he had CESD-10 measured five times respectively in 1999, 2001, 2002, 2004, and 2007, the corresponding time in reference to PD diagnosis will be 3 and 1 years before, the year at, and 2 and 5 years after PD diagnosis.

#### Covariates

Age (in years), sex (male/female), race (black/white), study site (Memphis/Pittsburgh), and education level (less than high school, or higher) were from baseline. Clinic visit year (i.e., 1 to 11), marital status (married/living as married vs. others), general health status (excellent to very good, good, or fair to poor), smoking status (current/former smoker vs. non-smoker), or use of antidepressants (yes/no) were time-varying and specific to each clinical visit. The study assessed the use of antidepressants (selective serotonin reuptake inhibitors, tricyclic antidepressants, MAO-B inhibitors, and miscellaneous antidepressants) in the past two weeks. There were no missing values in age, sex, race, and study site. If any of the time-varying covariates were missing at a visit, data from the nearest available visit were used. If missingness was throughout the entire study period, missing values were simply imputed as the values with the highest frequency in the data.

### Statistical analysis

After excluding participants who were missing CESD-10 scores (n = 2) at all visits, the current analyses included 79 PD cases and 2915 non-PD participants. Due to the relatively old age of our study participants and extended years of follow-up, longitudinal data missingness was likely informative of poor health and higher risk of death. We therefore analyzed data with the joint model to evaluate the changes in CESD-10 score over the years while accounting for the competing risks of non-participation and death [16].

To be specific, the model combined a linear mixed sub-model to evaluate the change trajectory of CESD-10 scores over time with the random intercept and the random slope of clinic visit year for each participant and a survival sub-model to account for the competing risks. We included in the linear mixed sub-model indicator variables for PD, the interaction between PD and year from PD diagnosis (hereafter referred to as timepoint), and clinic visit year (hereafter referred to as year). A quadratic term of clinic visit years was included to capture a non-linear changing trend. We also adjusted for baseline age, sex, race, study site, education, and time-varying covariates at various visits, including marital status, general health status, smoking status, and antidepressant use. The survival sub-model was fitted to evaluate the association between the longitudinal CESD-10 scores and time to non-participation and death, adjusting for PD status, PD-timepoint interaction, and the baseline values of all above-mentioned covariates.

Based on the jointly fitted linear mixed sub-model, at each time point, we calculated the expected CESD-10 scores of PD cases if they were still alive and had not developed PD (hereafter referred to as expected scores) by setting the indicator variable for PD status equals zero and holding all the other variables constant. We then calculated, for each time point, the mean difference between the CESD-10 score of a PD case and its expected value had the subject not developed PD, and derived the corresponding 95% pointwise Wald-type confidence intervals (CI). We conducted all analyses using R version 4.0.3 (R Foundation for Statistical Computing, Vienna, Austria). All statistical tests were two-sided with alpha of 0.05.

## Results

We presented baseline population characteristics of participants who developed PD in this cohort (cases) versus those who did not (non-PD participants) in **Table 1**. A total of 79 PD patients were identified, including 19 prevalent cases at year 1. Because of the narrow age range of the study population, PD cases and participants who did not develop PD during the follow-up did not differ in baseline age (74.0±2.7 vs. 73.6±2.9 years). Compared with non-PD participants, PD cases were more likely to be male (59.5% vs. 48.1%), white (74.7% vs. 58.2%), and married/coupled (73.4% vs. 56.1%), and to report high school or higher education (88.6% vs. 74.6%) and excellent-to-good overall health (94.9% vs. 83.9%) at enrollment. These two groups did not differ in study site or baseline CESD-10 score, antidepressant use, and smoking status.

**Table 1:** Baseline population characteristics of PD cases and non-PD participants.

Variable	PD cases	Non-PD participants	P-values ^a^
	(n = 79)	(n = 2915)	
Age in years, mean(SD)	74.0 (2.7)	73.6 (2.9)	0.20
CESD-10, mean(SD)	3.2 (3.1)	3.1 (3.4)	0.37
Sex, n (%)			
Male	47 (59.5)	1402 (48.1)	0.046
Female	32 (40.5)	1513 (51.9)	
Race, n (%)			
Black	20 (25.3)	1219 (41.8)	0.003
White	59 (74.7)	1696 (58.2)	
Site, n (%)			
Memphis	37 (46.8)	1463 (50.2)	0.56
Pittsburgh	42 (53.2)	1452 (49.8)	
Education, n (%)			
Less than high school	9 (11.4)	740 (25.4)	0.005
High school or higher	70 (88.6)	2175 (74.6)	
Marital status, n (%)			
Married/living as married	58 (73.4)	1634 (56.05)	0.002
Others ^b^	21 (26.6)	1281 (43.95)	
General health status, n (%)			
Excellent to Very good	38 (48.1)	1288 (44.2)	0.03
Good	37 (46.8)	1157 (39.7)	
Fair to Poor	4 (5.1)	470 (16.1)	
Smoking status, n (%)			
Current/former smoker	41 (51.9)	1641 (56.3)	0.44
Non-smoker	38 (48.2)	1274 (43.7)	
Depressive symptoms^c^, n (%)			
Yes	7 (8.9)	166 (5.7)	0.23
No	72 (91.1)	2749 (94.3)	
Antidepressant use ^d^, n (%)			
Yes	9 (12)	173 (6.3)	0.05
No	66 (88)	2562 (93.7)	

^a^ P-values were based on Mann Whitney U test and chi-square test for continuous and categorical variables, respectively.

^b^ Including never married, widowed, divorced, and separated.

^c^ Defined based on the 10-item Center for Epidemiologic Studies Depression Scale score of 10 or higher.

^d^ Any antidepressant (including selective serotonin reuptake inhibitors, tricyclic antidepressants, MAO-B inhibitors, and miscellaneous antidepressants) uses in the past two weeks.

**Fig 1:**
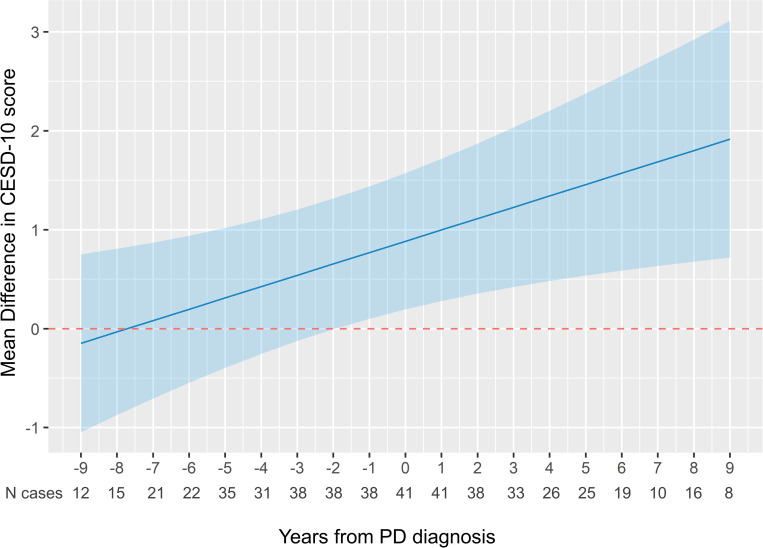
Mean difference in CESD-10 score before and after PD diagnosis. Y-axis represents the mean difference of CESD-10 score of a PD case versus its expected value had the participant not developed PD. The blue band represents the 95% confidence interval. Model adjusted for the linear and quadratic terms of clinic visit year, baseline age, sex, race, study site, and education, and the time-varying marital status, general health status, smoking status, and antidepressant use, and accounting for the competing risks of non-participation and death. PD: Parkinson’s disease; CESD: Center for Epidemiologic Studies Depression Scale.

Using the year of PD diagnosis as the reference point, we realigned data on CESD-10 score for up to 9 years before to 9 years after PD diagnosis (**Fig 1**). There was a monotonic and statistically significant trend of increasing mean difference of CESD-10 score between PD and their expected values over time (P = 0.002, **Fig 1** and **Table 2**). Starting approximately 7 years before PD diagnosis, the mean CESD-10 score became larger than its expected value had the subject not developed PD; however, the year-specific differences between PD and their expected values were not statistically different until approximately 1 year before diagnosis, which likely in part due to the small number of cases at each time point.

**Table 2:** Joint model estimates of the differences in CESD-10 score between a PD case and its expected value had the participant not developed PD ^a^.

CESD-10	Estimate (SE) ^b^	95% CI	P-values
**PD**	0.88 (0.35)	(0.20, 1.57)	0.002
PD*Timepoint ^c^	0.11 (0.05)	(0.03, 0.20)	0.002

Abbreviation: PD, Parkinson’s disease; CESD, Center for Epidemiologic Studies Depression Scale; SE, standard error; CI, confidence interval.

^a^ Adjusting for the linear and quadratic terms of clinic visit year, baseline age, sex, race, study site, education, and the time-varying marital status, general health status, smoking status, and antidepressant use, and accounting for the competing risks of non-participation and death. Random effects of the linear mixed sub-model include random intercept and random slope of clinic visit year for each participant.

^b^ The estimate for PD represents the mean difference of CESD-10 score at diagnosis; the estimate for the interaction term represents the mean annual change in the above-referenced difference.

^c^ Timepoint refers to the number of years from PD diagnosis.

## Discussion

In these PD patients with repeated assessment of depressive symptoms before and after PD diagnosis, we found a persistent trend of increasing depressive symptoms throughout the course of observation which became significantly greater than expected values about 1 year before diagnosis.

Depressive symptoms are common in PD, which may begin in its prodromal stage [3]. Multiple cohort studies have reported that depression or depressive symptoms predict the future risk of PD [17, 18], and one found that the association became stronger as it got closer to PD diagnosis [19]. These observations are consistent with the Braak hypothesis, which posits that PD Lewy pathology may affect the lower brainstem years before PD diagnosis [20]. The evidence, taken together, suggests that depression may be an integral part of prodromal PD development. As such, it has been proposed that research on key prodromal markers of PD, including depression, may help identify PD earlier in the disease process and aid better understanding of disease etiology [21], and inform novel treatment strategies [22].

Such an endeavor, however, depends on a good understanding of the temporal relationship of depression and other markers to PD clinical diagnosis, for example, when the symptoms begin to develop in prodromal PD and how they progress. This requires repeated assessments of these markers on a regular basis, years if not decades before PD diagnosis. Unfortunately, such data are rarely available. Prior studies have used alternative approaches. Based on retrospective recall, several case-control studies reported varying time intervals between depression and PD diagnoses ranging from <5 years [23] to 22 years [24]. Two other studies analyzed administrative claims data that recorded both depression and PD diagnoses. In the first study using data from the Registration Network Family Practices, Leentjens et al. reported the time span between the first depressive episode and PD diagnosis varied from one month to 36 years, with an average of 10.1 years [25]. In the second study using the UK primary care database, Schrag et al. reported depression incidence in future PD patients began to diverge from controls on average about 7–8 years before PD identification [26]. While these studies provide important clues as to when depression develops in prodromal PD, result interpretation is clearly limited by the study design and the fact that depression history was either recalled or assessed at a single time point, which prohibits the assessment of the dynamic nature of depressive symptomatology in prodromal PD. To our knowledge, only one previous study analyzed repeatedly measured depressive symptoms in prodromal PD. In the Rotterdam study, Darweesh et al. found that PD patients began to have significantly more depressive symptoms just before PD diagnosis [27].

The exact causes for depressive symptoms over the course of PD development and progression are likely complex and dynamic. They may arise as a manifestation of PD extranigral Lewy pathology, as the Braak hypothesis implies, or they may occur reactively to other PD symptoms and functional impairments as the disease progresses. Their contributions may also vary by the stage of the disease development and progression. For example, in prodromal PD, depressive symptoms may gradually develop as Lewy pathology develops in the lower brainstem such as locus coeruleus and lower raphe nuclei, followed by the substantia nigra, accompanied by neuronal losses [20]. In support, postmortem studies found markedly lower neuron density in these brain regions of PD patients with depression versus without [28–30]. Further, neurotransmitter imbalances and alterations in the noradrenergic, serotonergic, and dopaminergic systems have also been found in association with depressive symptoms in PD [31–34]. In addition, mechanisms such as neuroinflammation [35], hypothalamic-pituitary-adrenal axis dysregulation [36], and gut microbiota dysfunction [37] may also contribute to the development of depressive symptoms in PD. Over time, depressive symptoms may progress as PD pathogenesis further develops with the progression of other prodromal symptoms such as sleep disorders [38], poor olfaction [39, 40], and constipation [41]. As PD further progresses into the clinical stage, depressive symptoms may be further exacerbated by the shock of disease diagnosis, treatment [42], motor impairments and complications [43], fatigue [44], and cognitive decline [45]. While a mechanistic investigation is beyond the scope of this study, various mechanisms may interact to give rise to the dynamic presentation of depressive symptoms over the course of PD.

The current longitudinal study is very unique with repeatedly assessed depressive symptoms spanning over up to 9 years before to 9 years after PD diagnosis. Further, we conducted a comprehensive statistical analysis. In addition to known confounders, we controlled for the competing risks of non-participation and death, which is important in longitudinal analyses of older adults [16, 46], but was rarely considered in previous literature. In these PD patients older than age 70, despite a monotonic trend of increasing depressive symptoms starting in prodromal PD, the difference was modest and not statistically significant until approximately 1 year before diagnosis. This finding supports that from the Rotterdam Study [27] for closer proximity of the development of depression symptoms in prodromal PD to its clinical diagnosis. If this time interval is proven true, the potential utility of assessing depressive symptoms to identify prodromal PD patients may be limited.

This study has several limitations. First, study participants were relatively healthy and older at enrollment with a narrow age range; therefore, our findings may not be readily generalizable to the general PD population. Second, our sample size was relatively small with a total of 79 PD cases. Further, they were diagnosed at different time points during the follow-up, thus the actual sample sizes were varied at each specific time point in reference to PD diagnosis. Therefore, our results should be considered exploratory, which need to be confirmed in future larger studies with a similar design. Third, due to the small sample size at each time point in reference to the year of PD diagnosis and the lack of systematic data collection on antiparkinsonian treatments, we were unable to evaluate whether PD treatments might affect depressive symptoms after disease diagnosis. However, PD medication should not have any impact on the analysis of depressive symptoms in the prodromal stage of PD. Fourth, PD diagnoses were adjudicated retrospectively without collecting information on PD symptomatic onset, therefore, errors in diagnostic adjudication and time of disease identification are inevitable. Finally, although the CESD-10 is a validated screening tool for depressive symptoms, clinical diagnosis of depression requires comprehensive neuropsychological assessment.

In summary, in this longitudinal analysis of older PD patients with repeated assessments, we found depressive symptoms developed in prodromal PD and progressed in severity over time. However, its time course in prodromal PD may be shorter than previously thought.
